# A Randomized, Controlled Trial of Meditation for Work Stress, Anxiety and Depressed Mood in Full-Time Workers

**DOI:** 10.1155/2011/960583

**Published:** 2011-06-07

**Authors:** R. Manocha, D. Black, J. Sarris, C. Stough

**Affiliations:** ^1^Discipline of Psychiatry, Sydney Medical School, Royal North Shore Hospital, Sydney University, St Leonards, NSW 2065, Australia; ^2^Faculty of Health Sciences, Cumberland Campus C42, The University of Sydney, P.O. Box 170, Lidcombe, NSW 1825, Australia; ^3^Department of Psychiatry, The University of Melbourne, Melbourne, VIC 3121, Australia; ^4^NICM Collaborative Centre for Neurocognition, Swinburne University of Technology, Melbourne, Australia

## Abstract

*Objective*. To assess the effect of meditation on work stress, anxiety and mood in full-time workers. *Methods*. 178 adult workers participated in an 8-week, 3-arm randomized controlled trial comparing a “mental silence” approach to meditation (*n* = 59) to a “relaxation” active control (*n* = 56) and a wait-list control (*n* = 63). Participants were assessed before and after using Psychological Strain Questionnaire (PSQ), a subscale of the larger Occupational Stress Inventory (OSI), the State component of the State/Trait Anxiety Inventory for Adults (STAI), and the depression-dejection (DD) subscale of the Profile of Mood States (POMS). *Results*. There was a significant improvement for the meditation group compared to both the relaxation control and the wait-list groups the PSQ (*P* = .026), and DD (*P* = .019). *Conclusions*. Mental silence-orientated meditation, in this case Sahaja Yoga meditation, is a safe and effective strategy for dealing with work stress and depressive feelings. The findings suggest that “thought reduction” or “mental silence” may have specific effects relevant to work stress and hence occupational health.

## 1. Introduction

Health professionals, consumers, and patients are becoming increasingly enthusiastic about meditation; a survey of Australian GPs in 2000 found that almost 80% of respondents had recommended meditation to patients at some time in the course of their practice [1]. A nationally representative survey of US households in 1998 indicated that almost 1 in 5 consumers had used some form of “mind-body therapy” in the past 12 months, of which meditation was the commonest method [2]. While a survey of cancer sufferers in the UK found that meditative practices were the most popular complementary therapy used by this patient group [3].

The vast majority of research into meditation is focused on stress-related issues and indeed much of the enthusiasm amongst both health professionals and the general community is derived from these reports. Is meditation effective in reducing occupational stress, and if it is, is it more effective than placebo? Do different approaches to meditation have different effects? Canter explained that the majority of meditation research is characterized by poor methodological quality such that it was not yet possible to determine whether or not meditation is associated with a specific effect beyond that of placebo or simple rest [4]. Probably the most thorough and up to date review of meditation research was published in 2007 by a team led by Ospina, specifically contracted by the US Department of Health and Human Services to assess the evidence base [5]. They included both randomized and nonrandomized trials. In their assessment of more than 800 studies, they concluded

“Many uncertainties surround the practice of meditation. Scientific research on meditation practices does not appear to have a common theoretical perspective and is characterized by poor methodological quality. *Firm conclusions on the effects of meditation in healthcare cannot be drawn based on the available evidence*.”

A handful of key methodological and conceptual problems were identified that, if addressed, would significantly advance our understanding about meditation's potential role in clinical practice. Two problems of particular importance are, first, the need for strategies to control for nonspecific effects associated with any meditation-like intervention and, second, greater clarity in defining meditation. 

Stress is currently understood in terms of an individual's sense of control over the events and symptoms in one's life [6]. When individuals believe that they can control negative events, they cope better and experience less stress. It is commonly defined as “a particular relationship between the person and environment that is appraised by the person as taxing or exceeding his or her resources and endangering his other wellbeing” [7]. Stress is associated with physiological hyperarousal, negative cognitions, and negative mood and has been associated with a wide variety of physical and mental health problems. The relationship between psychosocial stress and cardiovascular disease, for example, is becoming increasingly significant to clinicians [8, 9]. A 2006 insurance survey reported that seven million Britons experiencing stress related symptoms sufficient to compel them to seek medical attention [10]. Studies estimate that 50–70% of general practice consultations feature stress-related issues [11]. The Bristol Stress and Health Study assessed 17,000 workers and found that approximately 20% of respondents experienced very high or extremely high levels of stress at work and that this stress was associated with negative effects on physiology, mental performance, and risk of work place accidents [12].

Individually orientated interventions for stress such as meditation are simple yet potentially effective health promotional strategies [13]. As a result, they are becoming increasingly popular within organizations [14]; however, these interventions have to date not been rigorously evaluated. In fact, only a small number of RCTs of meditation for work stress have been reported in the literature [15–22] and none so far provide convincing evidence for a specific effect. Meditation is commonly thought to reduce stress by a combination of two pathways. First, by reducing somatic-arousal (physiological effects) [23] thereby reducing reactivity of the individual to environmental stressors, and second, by altering the individual's cognitive appraisal of and perceived self-efficacy with regard to stressors [24]. The cognitive-behavioral effects are thought to result from the meditator's increased awareness of how thoughts and emotions arise in response to various environmental events, thereby allowing the meditator to achieve more veridical perception, reduced negative affect, and improved vitality and coping [25]. 

The RCT reported here was thus designed to address two interconnected questions: first, and primarily, whether or not meditation is a useful strategy for dealing with work-related stress, anxiety, and depression (a common problem encountered in primary health and a major public health concern); second, whether or not, the use of appropriate controls, randomization, blinding, and a “mental silence-” rather than “relaxation-” orientated approach to meditation can help answer the question about whether or not meditation might have a specific and clinically useful effects.

## 2. Methods and Materials

### 2.1. Overview

We designed an 8-week, 3-arm, parallel randomized controlled trial which compared a “mental silence” orientated style of meditation called sahaja yoga to a “relaxation-oriented” meditative control and a waiting list (no treatment) control. The trial was conducted from 2002 to 2003 in the CBD of Sydney, Australia. The study was approved by the South Eastern Area Health Service Ethics Committee (NSW dept of health) and registered (Trial Registration Current Controlled Trials number ISRCTN46301450).

### 2.2. Participants

Eligibility criteria were full-time employment (more than 30 hours per week), willing to commit to the instructional program and twice daily practice at home, nonsmokers, imbibing less than 2 units of alcohol daily, free of serious psychological/psychiatric/medical morbidity, not using other stress management strategies (including other meditation techniques, methods of relaxation, or participation in any other organized stress management programs in the past 12 weeks), have experienced no recent major life events (such as bereavement/major illness in immediate family, moving house, recent divorce or relationship breakdown), not using recreational drugs, willing to fill out a questionnaire battery before and after the program. Participants were recruited by advertising in local newspapers and other popular media. 

### 2.3. Interventions

For both active intervention groups, the intervention period was 8 weeks, involving 1-hour evening sessions twice weekly. Participants were required to practice twice daily at home for 10–20 minutes each time. Compliance with this regime was reinforced at each instructional session. Instructors for both active groups were health professionals who were also experienced and qualified meditation instructors. 

The mental silence meditation (MSM) group was taught to elicit a state of mental silence or “thoughtless awareness”. The technique is based on Sahaja yoga, a noncommercial, “classical” understanding of meditation. The main technique employs a simple series of silent affirmations based on a traditional understanding of yogic psychophysiology [26]. Subjects were encouraged to meditate while sitting quietly in a chair or in a comfortable position that facilitated their meditation experience. The mental silence experience is attainable in several different ways, all of which converge on the central principle that when the attention is focused on the experience of the absolute present moment, rather than events of the past or future (even the most recent past or imminent future), thinking activity ceases despite remaining fully alert and in control of one faculties. At first, this cessation of mental activity is short lived but with practice it can be drawn out into a continuous, enjoyable, experience which meditators consistently describe as peaceful and stress-free [27]. The meditation techniques taught to participants were simple strategies aimed at facilitating this experience. Affirmations, breathing techniques, and attention focusing exercises were taught in a graded fashion with the emphasis placed on achieving and maintaining a sustainable state of “mental silence” (Sanskrit “nirvichara samadhi” or “*thoughtless awareness*”). Each week informal feedback was sought by instructors regarding each participants' progress with regard to this experiential dimension. 

The nonmental silence, relaxation control group (RC) was a generic meditative technique based on the “relaxation response” [28]. It was developed by a professional meditation instructor specifically for the study. Subjects were instructed to sit comfortably, breathing regularly and commence their meditation by reflecting on the day's events. The instructor sought feedback each week from participants in order to ensure that the meditative style was adhered to. This intervention was thus designed to control for nonspecific effects associated with reduction in physiological arousal (i.e., “rest”) as well as other nonspecific factors such as therapeutic contact, credibility, and expectancy associated with any behavioral intervention. 

The no-treatment (NT) group was comprised of subjects who were told that they were on a waiting list to be admitted into one of the meditation groups at a later date. They were not told that they were a control group. The waiting NT group was included to control for practice effect associated with the psychometric questionnaires, regression to the mean and other nonspecific effects [29].

### 2.4. Randomization and Blinding

A research assistant located separately from the main investigators randomly allocated each subject from each round of recruitment to one of the three groups using a blindfolded lottery allocation system. The subject was notified of their allocation by the assistant, and this was not disclosed to the investigators. Participants and instructors were blinded to the complete hypothesis of the trial, were not informed about what methods were being used in the comparison groups, and were instructed not to disclose information about the methods used in their classes to other trial participants or the investigators. The investigators, data entry personnel, scorers, and statistician were also blinded to group allocation. The two meditation interventions were structured identically such that nonspecific factors such as credibility, expectation, and demand characteristics were matched as closely as possible. Classes for both intervention groups were conducted at the same institutional location, in similar rooms, at the same time of day, with similar support materials; instructional sessions were of equal duration with equivalent periods between interventions.

### 2.5. Measures

Baseline assessments were done prior to randomization and at completion upon week eight. All consenting potential participants were invited to an evening information session where the basic principles of the study were outlined, including inclusion and exclusion criteria. Those participants who decided that they were able to satisfy these criteria were invited to remain and fill out the baseline questionnaire battery. Any questions or difficulties with the questionnaire were directly addressed by researchers who were also on site at the time of the briefing/baseline questionnaire session. Within one, week participants were allocated to their treatment group and the instructional program commenced. Recruitment was done in batches in such a way that the information/baseline questionnaire sessions were not conducted until a minimum number of volunteers had accumulated, usually at least 30 per batch. Postintervention assessments were similarly conducted between 5 and 7 days after the final instructional session, specifically in order to avoid biasing that may arise from acute effects of the intervention. 

Consenting participants were assessed on several valid measures of stress, anxiety, and mood involving: the Psychological Strain Questionnaire (PSQ), an accepted measure of work stress and part of the larger Occupational Stress Inventory (OSI) [30]. The PSQ focuses specifically on the subjective “work stress” experience, whereas the larger parent questionnaire assesses environmental stimuli and coping mechanisms as well; the State component of the State/Trait Anxiety Inventory for Adults (STAI) [31]. The state sub-scale of the STAI has been widely used for the assessment of general anxiety but does not restrict itself to anxiety at work. We elected not to use the trait subscale as this was a short-term treatment programme, and we did not anticipate to observe changes in this dimension; and the depression-dejection subscale of the Profile of Mood States (POMS) to assess depressive symptoms [32]. The POMS does not restrict itself to work stress but addresses general emotional states. The GHQ28 was used to assess the mental health profile of our sample before treatment. The SERCIS study used this instrument to assess the mental health profile of an Australian sample representative of the general population [33]. The GHQ, in its various forms, has been demonstrated to be a reliable estimator of nonspecific psychological distress and demoralization [34].

### 2.6. Data Analysis

Data was analyzed with the intention of treating basis. Data for participants lost to followup was estimated using the last observation carried forward (LOCF) method. SPSS Version 14.0 was used for analyses. Differences in pre- and postscores were calculated for the primary outcome measures. If the differences were normally distributed, a one-way ANOVA was used to compare the mean differences. For skewed data, a median test of significance was used to compare frequencies of values above and below the median in the three groups. A meaningful change in any of the chosen measures was classified as more than 30% improvement (a relatively high threshold) as a positive “improved” clinical response. Those whose score declined by 15% or more were classified as “declined”. Multiple logistic regression was used for improved/declined in the outcome measures. Demographic data were included in the logistic regression model if they were associated with an improvement with *P* < .25. Work-related variables including classification of occupation were included in a covariate analysis of work stress variables. 

## 3. Results

### 3.1. Sample

**Figure 1:**
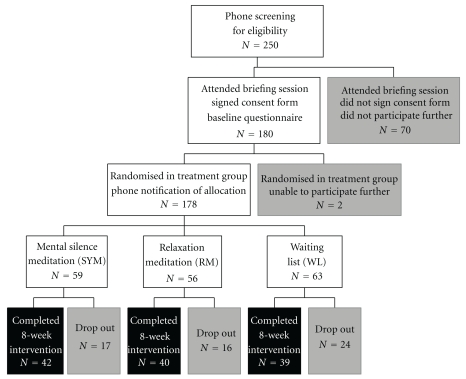
Consort diagram for work stress study.

In total, 250 people fulfilled phone screening criteria and attended an information session about the trial (see Figure 1). Of these, 180 decided to participate and were randomized to one of three groups. Two people withdrew shortly after randomization, prior to the first class. The dropout rate at completion of the study was 32% with no significant differences between the groups (*χ* ^2^ = 1.65, *P* = .44). The groups had similar characteristics at baseline (see Table 1). Average compliance rate was the same in both intervention groups (81% attending maximum possible classes). Dropouts tended to occur earlier in the MSM group (after 37% of classes were attended) compared to the RC group (after 50% of classes attended) strongly suggesting that credibility and expectancy was very similar in the two active intervention groups. The GHQ 28 baseline assessment indicated that the participants as a whole were experiencing considerably more mental distress than the general population. Using the scoring system recommended by its developers, it is generally agreed that a GHQ score of 5 or more indicates high risk of mental health morbidity. The mean score of the reference population from the SERCIS survey was 2.45 (95% CI: 2.3–2.61) [35]. The mean baseline score of our sample was 7.5. While the SERCIS survey found that 19.5% of the general population had a score indicating mental health morbidity, our sample had 47% of participants in the same category.

### 3.2. Outcomes on Stress, Anxiety, and Mood

**Figure 2:**
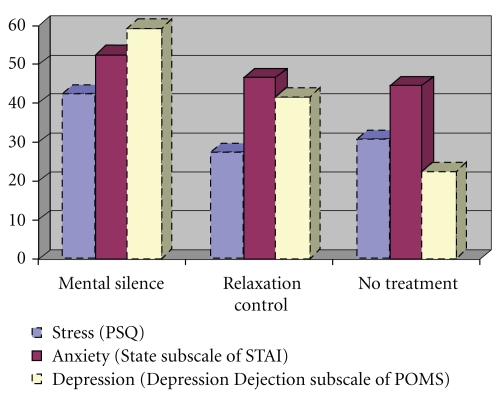
Responder rates for work-related stress, anxiety, and Depression*. A percentage improvement of >/=30% was classified as a positive response at week 8.

After adjusting the data for the primary outcomes on the basis of intention to treat (LOCF), there was a statistically significant improvement for the MSM group (see Table 2) compared to both the RC and no treatment groups in the median differences for occupational stress symptoms (*P* = .026) on the PSQ, and depressive symptoms (*P* = .019) on the DD subscale of the POMS. While an improvement in median difference on STATE anxiety for the MSM group was noted, it was not statistically significant (*P* = .209) within the intention to treat analysis (Figure 2).

The percentage changes in scores for the three primary outcomes were categorized into “1” for improvements of 30% or more and “0” for changes less than 30%. There was a statistically significant improvement in occupational stress symptoms (*P* < .05; PSQ) and depressive symptoms (*P* < .001; POMS DD subscale). In the multiple logistic regression analysis for occupational stress symptoms, the occupation variable was included as a covariate. Comparing the no-treatment group with the MSM group showed a significant improvement in favor of the active intervention (*P* = .034, OR = 2.64, 95% CI: 1.22–5.68). There was no significant improvement in the RC group compared to the NT group (*P* = .546, OR = 1.266, 95 CI: 0.589–2.724). There was no association between improvement in PSQ and occupation (*P* = .999, OR = 1.00 95% CI: 0.491–2.033) (Table 3).

In the multiple logistic regression analysis for depressive symptoms (data not shown), sex was included as a covariate. Comparing the NT group with the MSM group showed a significant improvement in favor of treatment (*P* < .001, OR = 5.27, 95% CI 2.38–11.69). There was also a significant improvement in the RC meditation group compared to the NT group (*P* = .029, OR = 2.441, 95 CI 1.10–5.43). There was no association between improvement in depressive symptoms and sex (*P* = .373, OR = .701 95% CI: 0.320–1.534). 

## 4. Discussion

This study has a number of strengths that assert progress in the field of meditation research. First, the use of a large sample, and rigorous methodology, particularly the efforts taken to exclude the effects of nonspecific factors is a notable methodological strength. This is one of the largest RCTs to make an earnest attempt to control for nonspecific effects and one of the only independent RCTs to compare two different conceptual understandings of meditation. Second, in this study there was no evidence of adverse effects associated with either intervention since both intervention groups generated significantly fewer negative responders than the untreated group. This is an important though often neglected consideration. Third, this study provides evidence to suggest that a “mental silence” definition of meditation is more likely to be associated with specific benefit. The implications of this third point are particularly fascinating, and we discuss some of them below. 

A differential effect across the two intervention groups compared to nontreatment was found in this study. Our findings indicate that the mental silence-orientated approach is specifically effective in reducing work-related stress and depressive feelings. This is the first RCT of this approach to meditation for occupational stress to clearly demonstrate a specific effect in comparison to credible controls (waitlist nontreatment and relaxation). Similar findings were observed in an RCT comparing the same approach to meditation to a standardized stress management intervention for sufferers of moderate to severe asthma (on prestabilized treatment but who remained symptomatic). It demonstrated significantly greater improvements in a number of important subjective and objective outcome measures associated with meditation [36]. 

A field study in which 293 medical practitioners were taught a meditation skill based on Sahaja yoga for the enhancement of psychological well-being made a number of important observations with regard to the relationship between mental silence and the study outcomes [27]. The relationship between participants' self-reported experience of “mental activity/silence” and their self-reported experience of “calm/peaceful” and “tension/anxiety/stress” was strong and highly significant, such that the more that participants' mental activity moved toward the silent state, the more calm/peaceful (*r* = 0.78, *P* < .001) and the less tense/anxious/stressed they felt (*r* = 0.70, *P* < .001). In the diary card data, a significant relationship between self-rated mental silence and K10 score such that a higher self-rated score of mental silence was associated with a lower level of psychological distress (i.e., a lower K10 score). Among those GPs who regarded the intervention as highly effective, there was a significant positive relationship between the change in mental silence rating and change in K10 score. Taken together, (this study and Manocha's field study) suggest an effect not simply attributable to relaxation or placebo, indicating that “reduction of thought activity” has particular effectiveness for the reduction of stress and stress-related illness. 

A fundamental challenge for those who design RCTs of meditation is how to develop the behavioral equivalent of a “sugar pill”. In this study, we explore an innovative strategy to address this challenge. Since reviews such as Ospina's and other thorough examinations of meditation published in the literature suggest that the “relaxation” model of meditation generates a predominantly nonspecific effect then, rather than using it as an intervention, we have in this study used “relaxation” as a control. In the context of this study, by comparing the “relaxation” model of meditation to a more classical Eastern “mental silence” model this study might not only be understood as a trial that controls for the important nonspecific effects (placebo, credibility, activity, and physiological dearousal associated with relaxation) but also as a head to head comparison of two differing conceptualizations of meditation. In this scenario, despite both approaches being “meditative”, the approach that emphasized the experience of mental silence demonstrated an effect greater than the one that emphasizes relaxation. 

Conventionally, the stress reducing effects of meditation have been ascribed to meditation's ability to reduce physiological arousal. Following this line of thinking, the effects observed in this study may have occurred because mental silence-orientated forms of meditation simply reduce physiological arousal more effectively than relaxation-orientated approaches to meditation. Alternatively, current theories of stress might explain the observed changes as arising from the possibility that mental silence may more effectively facilitate greater awareness by reducing distracting and unnecessary mental activity thereby facilitating better veridical perception, reduced negative affect, and improved vitality. This contrasts with methods of meditation that emphasize relaxation, or other models of meditation that do not involve mental silence. 

There is some experimental data suggesting that mental silence-orientated approaches to meditation might act via pathways that are different to simple relaxation. For example, Aftanas has conducted neurophysiological trials of the same mental silence-orientated meditation, assessing EEG changes in advanced meditators. The research revealed that the practice was associated with reproducible brain electrical changes, and that these patterns correlated strongly with the specifically defined, self-reported experience [37, 38]. A small study in which the same approach to meditation was compared to rest demonstrated that while those who “rested” manifested skin temperature increases consistent with the “relaxation response” paradigm, those who meditated in “mental silence” manifested skin temperature reduction. Yet the heart rate changes in both groups were not significantly different. Interestingly, the degree of skin temperature reduction in the meditation group correlated highly with meditator's self-reported experience of mental silence [39]. The skin temperature changes suggest that a potentially unique fractionation of the relaxation response occurs in association with the mental silence experience. This implies that the mental silence-orientated conceptualization of meditation may be associated with specific physiological changes. Perhaps these changes are responsible for the specific effects observed in this study. Future studies of this approach to meditation should therefore correlate clinical and behavioral changes with convention measures of arousal.

Until 2006, the U.S. National Center for Complementary and Alternative Medicine (NCCAM) defined meditation as “a conscious mental process that induces a set of integrated physiological changes termed the relaxation response” [40]. Remarkably, however, in 2006 the NCCAM reviewed its definition of meditation, describing a new central feature: “In meditation, a person learns to focus his attention and *suspend the stream of thoughts that normally occupy the mind*.” [41] The fundamental change in emphasis from the physiology of rest (a Westernized understanding of meditation) to the experience of “suspension of thought activity” (a more classical eastern idea of meditation) raises an important question about whether or not this shift in conceptualization has practical and clinical significance. Our study throws some important empirical light on these theoretical and philosophical shifts. On a more theoretical level, meditation is popularly perceived as having specific effects. In fact historical tradition, especially Eastern tradition, asserts that meditation has a unique effect and yet the scientific evidence, based mainly on studies of Westernized models of meditation, does not agree with these perceptions. The outcomes of this trial suggest that one way to resolve this conundrum may be to propose a definition of meditation based on the “experience of mental silence”.

We do acknowledge limitations to this study. Our primary research question was whether or not mental silence meditation had a specific effect on work stress and this is best assessed at the postintervention point; therefore, this trial did not incorporate a follow-up assessment. In light of the outcomes of this study, future studies warrant a follow-up assessment strategy to assess whether participants continue using the intervention and the degree to which the benefits are maintained. The use of instruments such as the PSQ, DD, and STATE in our study may be considered by some as not adequately objective, but it should be noted that the use of such measures is currently considered to be both a reliable and standard approach to studying the effects of interventions for work stress. There is good evidence that these measures are clinically useful and reliable and in fact, although more objective measures might be more desirable in studies like this, there is currently no agreement amongst work stress researchers about which objective measures are *both* reliable *and* feasible for use in field studies. 

The use of intention to treat analysis in this study is likely to give a very conservative understanding of the independent variable's impact. The dropout rates in our trial were similar to other trials of meditation for work stress and meditation trials in general. The dropout rates in the two groups were not significantly different, and there were no significant differences between the dropouts and finishers in baseline and demographic data suggesting that their exit did not introduce any major selection bias. Compliance measures would be theoretically useful as a covariate in the analysis of the outcome data. We did not, however, assess home compliance directly because our experience in pilot studies was that assessments such as daily practice diaries were not sufficiently reliable. We did not assess credibility of the two active interventions. This is a potential drawback however as both were legitimate interventions in their own right. Moreover, the fact that dropout rates in both groups were not different strongly suggests that both interventions were sufficiently and similarly credible. This trial required subjects to attend after normal working hours at a site separate from their workplace, however, future trials that are well integrated into daily activities within an organization may generate significantly lower dropout rates. Finally, the recruitment of participants working in the CBD of Sydney in the study through newspapers and other media means that inferences from the study can only be made to the population defined as responding to the media of an industrialized area with a mainly Caucasian population.

## 5. Conclusion

This study provides preliminary evidence to support the use of a mental silence form of meditation called Sahaja Yoga to reduce work stress and depressed mood. While the results are encouraging, further research is now required to validate and explore these findings. Given the low-cost, noncommercial nature of the intervention, and the low risk of adverse effects it would not be unreasonable to suggest that this meditation would be useful as a health enhancing strategy with potential for significant socioeconomic benefit to individuals and society. 

This trial provides initial evidence indicating that there are measurable, practical, and clinically relevant differences between two differing conceptualizations of meditation. On a practical level Sahaja Yoga, and by inference, possibly any meditation technique that is specifically mental-silence oriented, is safe and effective as a general intervention strategy for dealing with work stress. It suggests that those forms of meditation that emphasize “thought reduction” or “mental silence” may have specific effects beyond simple relaxation techniques that may be relevant to health care. The fact that this trial was a field study of a group with demonstrably higher levels of psychological distress when compared to available population norms strongly suggests that the intervention is feasible and relevant in the “real world”.

## Figures and Tables

**Table 1:** Comparison of demographic data and primary outcome measures at baseline.

Demographic	Mental silence	Relaxation	No treatment	*P* value
Mean age (95% CI)	42.5 (39.8–45.2)	41.4 (38.9–44.0)	42.3 (39.4–45.2)	.835^a^
% White collar worker	76%	80%	64%	.123^b^
% > secondary education	46%	57%	45%	.501^b^
PSQ (95% CI)	100.5 (94.6–106.3)	100.4 (94.6–106.3)	99.9 (92.8–106.9)	.988^a^
STATE (95% CI)	41.0 (38.0–44.0)	41.3 (38.5–44.1)	40.3 (37.8–42.9)	.869^a^
DD (95% CI)	14.4 (11.2–17.6)	14.0 (12.0–17.7)	12.3 (9.8–14.8)	.384^a^

^a^one-way ANOVA; ^b^ *χ* ^2^ test; PSQ: Psychological Strain Inventory; STATE: State/Trait Anxiety Inventory for Adults; DD: Depression Dejection (Subscale of POMS).

**Table 2:** Baseline and week-8 median differences on primary outcome measures.

Outcome measure	Mental silence	Relaxation	No treatment	*P* value
Median difference PSQ	37.0	22.3	17.5	.026^a∗^
Median difference STATE	−15.0	−8.5	−9.0	.209^a^
Median difference DD	−3.0	0.0	0.0	.019^a∗^

^a^one-way ANOVA; *Significant *P* < .05; PSQ: Psychological Strain Inventory; STATE: State/Trait Anxiety Inventory for Adults; DD: Depression Dejection (Subscale of POMS).

**Table 3:** Responders at completion at week 8 on primary outcomes^*≠*^.

Outcome measure	Mental silence	Relxation	No treatment	*P* value
% improving 30% + in PSQ	42.4%	27.1%	30.6%	.045^a∗^
% improving 30% + in STATE	52.5%	46.4%	44.4%	.651^a^
% improving 30% + in DD	59.3%	41.1%	22.2%	<.001^a∗∗^

^a^one-way ANOVA, *Significant *P* < .05, **Highly significant *P* < .01, ^*≠*^A percentage improvement of >/=30% was classified as a positive response; PSQ: Psychological Strain Inventory; STATE: State/Trait Anxiety Inventory for Adults; DD: Depression Dejection (Subscale of POMS).
